# The impact of armed conflict on utilisation of health services in north-west Syria: an observational study

**DOI:** 10.1186/s13031-021-00429-7

**Published:** 2021-12-14

**Authors:** Abdulkarim Ekzayez, Yasser Alhaj Ahmad, Hasan Alhaleb, Francesco Checchi

**Affiliations:** 1 Research for Health System Strengthening in Northern Syria (R4HSSS), War Studies Department, Faculty of Social Science and Public Policy, King’s College London, Room K6.08, King’s Building, Strand Campus, Strand, London, WC2R 2LS UK; 2 Syria Public Health Network (SPHN), London, UK; 3 Health Programme, Save the Children International, North West Syria Response, Antakya, Turkey; 4 Monitoring and Evaluation Department, Syria Relief Charity, Antakya, Turkey; 5 Department of Infectious Disease Epidemiology, Faculty of Epidemiology and Population Health, London School of Hygiene and Tropical Medicine, London, UK

## Abstract

**Background:**

Armed conflicts are known to have detrimental impact on availability and accessibility of health services. However, little is known on potential impact on utilisation of these services and health seeking behaviour. This study examines whether exposure to different types of war incidents affected utilisation of key health services—outpatient consultations, antenatal care, deliveries, and C-sections, in conflict affected areas of north west Syria between 1 October 2014 and 30 June 2017.

**Methods:**

The study is an observational study using routinely collected data of 597,675 medical consultations and a database on conflict incidents that has 11,396 events. Longitudinal panel data analysis was used with fixed effect negative binomial regression for the monthly analysis and distributed lag model with a lag period of 30 days for the daily analysis.

**Results:**

The study found strong evidence for a negative association between bombardments and both consultations and antenatal care visits. The monthly Risk Ratio was 0.95 (95% CI 0.94–0.97) and 0.95 (95% CI 0.93–0.98); and the cumulative daily RR at 30 days was 0.19 (95% CI 0.15–0.25) and 0.42 (95% CI 0.25–0.69) for consultations and antenatal care respectively. Explosions were found to be positively associated with deliveries and C-sections. Each one unit increase in explosions in a given month in a given village was associated with about 20% increase in deliveries and C-sections; RR was 1.22 (95% CI 1.05–1.42) and 1.96 (95% CI 1.03–3.74) respectively.

**Conclusion:**

The study found that access to healthcare in affected areas in Syria during the study period has been limited. The study provides evidence that conflict incidents were associated negatively with the utilisation of routine health services, such as outpatient consultations and antenatal care. Whereas conflict incidents were found to be positively associated with emergency type maternity services—deliveries, and C-sections.

## Introduction

The detrimental impact of armed conflict on health is not limited to deaths, injuries and disability; conflict also bears indirect health effects through a variety of risk factors. Attacks on healthcare and other disruptions to health systems reduce access to curative and preventive services [1]. Current wars tend to be protracted and increasingly take place in urban settings, which can amplify their health consequences [2]. It is estimated that armed conflicts kill around 133,750 people every year [3], but this estimate does not account for indirectly attributable mortality. Warring parties may deliberately seek to damage and curtail access to civilian health services, or may do so collaterally due to their military tactics. In either case the laws of war are contravened, but, aside from deaths and damage directly resulting from attacks on health services, the full effects of such actions are not easily quantifiable. Stronger evidence could better illuminate the scale of the problem, support memorialisation of wars, inform civilian protection and potentially aid prosecution of war crimes.

More than ten years of conflict in Syria has had a devastating impact on civilians, infrastructure and services, with more than half a million deaths [4] and half of the entire population displaced either internally or to neighbouring countries [5]. What started as peaceful demonstrations in March 2011 has turned into a proxy war with complex regional and international dimensions [6], dividing the country into different areas of control. As of summer 2021, Assad regime controls Damascus and most central, coastal and southern areas, the Self Administration and the Kurdish-majority Syrian Democratic Forces controls the northeast, and opposition armed groups controls the north west, the latter with a population of approximately 3.5 million as of April 2021 [5].

The health system in northwest Syria was severely affected by the conflict. The withdrawal of the Damascus ministry of health starting from 2012 from all opposition-controlled areas, shortages in resources including health workforce, attacks on healthcare, and the lack of central health authority to coordinate health interventions are all some of the challenges faced by this health system [7]. Attacks on healthcare have been a pre-eminent war tactic that was often used in these areas [8–11]. As of June 2021, some 600 attacks on 350 health facilities, most perpetrated by the Government of Syria (GoS) and its allies, had been recorded [12], and 27% of Syrians lived in areas where health workers are absent [13].

As part of the cross-border humanitarian response in northwest Syria, local and international Non-Governmental Organisations (NGOs) started to support health interventions since 2012, followed by UN agencies after the UN security council resolution 2165 in July 2014 [14]. Considering security threats, the health response is heavily dependent on local actors who usually partner with international NGOs to deliver health programming. Between 2014 and 2017, the charity Save the Children International (SCI) supported a network of eight health facilities in north west Syria in partnership with two Syrian NGOs, Syria Relief and Shafak. The areas served by these seven primary healthcare facilities and one maternity hospital were also receiving health services from other humanitarian actors; however, for some types of services, such as family planning and C-sections, the number of other providers was very limited during this period.

We analysed service data from this network, in combination with population and conflict incident data, to estimate coverage of the studied health facilities and to quantify the association of attacks and military operations on health service utilisation.

## Methods

### Study population and period

The SCI project consisted of seven Primary Health Care (PHC) facilities providing outpatient consultations, first aid, dressing and suturing, the Minimum Initial Service Package (MISP) for reproductive health, referrals, and Basic Emergency Obstetric Care (BEmOC); and one maternity hospital offering Comprehensive Emergency Obstetric Care (CEmOC). The project had a theoretical catchment population of about 250,000, spread across a large portion of Idleb governorate, rural areas of western Aleppo governorate and northern Hama governorate. This analysis covers the period October 2014 to June 2017.

### Data sources

The SCI project developed a bespoke Health Management Information System (HMIS) using a Microsoft SQL server, used to collect, manage and archive individual electronic patient-provider contact records. Four authors (AE, YHA, and HA) were involved in managing this project and developing the HMIS. Unique identifiers were deleted after data aggregation. We excluded records prior to October 2014 as these were collected using a preliminary version of the HMIS, and considered data up to 30 June 2017. The analysis dataset comprised 637,824 patient-provider contacts.

No consolidated database of conflict incidents at the community level was readily available for the study period. We thus constructed such a dataset by systematically consulting two main sources: “Shaam News Network” [15] and “Ugarit News”: both are local news agencies that feature daily narrative field reports summarising all war incidents in Syria. We verified incidents with casualties by triangulating the information with three other sources: the Syrian Observatory for Human Rights, Al Jazeera and BBC news. More than 1500 daily reports were reviewed for the period between 1 October 2014 and 30 Jun 2017. We extracted meta-data (date, location, type, number of people killed) on all (n = 11,396) war incidents taking place within the project catchment area.

Lists and spellings of settlements (e.g. village names) patients originated from were matched against a master geographic dataset maintained by the United Nations Office for Coordination of Humanitarian Affairs [16]. We excluded from analysis 296/882 ‘settlements’ mentioned on the patient database that could not be matched to this master dataset (these may in fact have been neighbourhoods or streets). We also computed line-of-sight- distances between settlements and their closest health facility based on both locations’ coordinates: 332 settlements > 15 km away from any health facility were also excluded based on average walking distance to access health facilities as per UNHCR standards for primary health care coverage [17], and based on authors’ experience of travel time limits to realistic accessibility in the Idlib context. In practice, the above exclusions only reduced the total number of patient records from 637,824 to 583,781, as the excluded settlements accounted for very few patients, suggesting most were outside the effective catchment area of the SC project. Lastly, settlements were allocated to the catchment of the health facility to which they sent most patients.

Lastly, it was challenging to get accurate estimates for population figures. The National Population Monitoring (NPM) surveys were used as population denominators [18]. The NPM was a joint initiative by the International Organization for Migration and OCHA in which the populations of most communities in Syria were estimated on a monthly basis starting from December 2015, adjusting for displacement. In the absence of displacement data before this period, the first available (December 2015) estimate was also applied to the period from October 2014 to November 2015.

### Study outcomes

The primary outcome we looked at was new outpatient consultations. Antenatal care visits, in-facility deliveries and caesarean sections were secondary outcomes. The choice of these outcomes was mainly opportunistic based on data availability.

### Exposures: conflict incidents

War incidents were classified as (i) bombardment, defined as any bombing or shelling, either aerial or ground; (ii) explosion, defined as any explosion, detonation or burst caused by explosive devices, cars or remnants; (iii) clashes, defined as any active fighting or clash between any of the warring parties.

### Inclusion criteria

All the health facilities, except the maternity hospital, were closed for a couple of weeks in October 2015 due to security threats faced by Save the Children. Also, the maternity hospital was hit by an airstrike on 29 July 2016, and it was closed for less than two weeks following this attack. For these periods, data from the closed health facilities were excluded from the daily analysis. However, it was not excluded from the monthly analysis considering that there were only two incidents of facility closure none lasted for more than two weeks.

For conflict incidents, the database includes variables that indicate scale of incidents, such as number of casualties and injuries. However, considering the unreliability of this type of data we have not used these variables in our analysis.

### Patient and public involvement

The study is an observational study of a routinely collected health data, and hence patients were not involved in the study. However, all the data was anonymised and aggregated on facility level before starting the analysis. The findings presented publicly do not include any individual or even facility-level data. Therefore, additional patient consents were not sought; however, further ethical oversight from the organisations involved in running the health facilities was sought.

During the design phase of this study, the organisations that were running the health facilities; Save the Children, Syria Relief and Shafak Syria; were all consulted. They all agreed on the importance of the study questions and their policy and advocacy implications. They also reviewed the proposed methodology, data management plan, and analysis plan to ensure ethical considerations are adequately addressed.

### Statistical analysis

The statistical analysis started with an exploratory and descriptive analysis, data integrity checks, and trend analysis followed by two causal modelling:

- Monthly analysis (village—month panel data): A negative binomial regression for longitudinal panel data (monthly outcomes grouped by village).
- Daily analysis (village—day panel data): A negative binomial distributed lag model for longitudinal panel data (daily outcomes grouped by village) with a lag period of 30 days. We opted for a 30-day lag period for the daily analysis to be comparable to the monthly analysis.

Some other regression models, such as Poisson and Zero Inflated Negative Binomial, were explored and excluded given the nature of the data and using Likelihood Ration Tests and multicollinearity checks. As Gardner and others suggest, Poisson model for count data can be misleading considering that individual observations are usually over-dispersed than is assumed by the model, and negative binomial regression represent a valid alternative [19]. The model approach took into consideration that the data is count clustered by village, and the data is dispersed and skewed to the right with a high proportion of zero values. Both models used fixed effects, an offset of the estimated population of each village and the following predictors:

- The three conflict exposures (bombardment, explosions and clashes);
- whether the day/the month falls within Ramadan;
- which season the day/the month falls within;
- distance from the closest health facility, initially as a linear term;
- and, for the daily model: whether the day is Friday.

We used fixed effect models with an assumption that the observations are independent between and within villages. This assumption was based on direct observations during the period of data collection which indicate frequent and massive changes of the study population with relation to population movements and displacement, areas of military control, road accessibility, and availability of health services. All these factors contributed to a greater variation between villages and even within villages leading us to assume independence of the observations.

For confounders, the authors’ own medical work inside Syria suggested that healthcare utilisation decreases during Ramadan. Internal clashes between local armed groups in northwest Syria also tend to decrease during the holy month. The same applies for extreme weather, either in summer or winter when both health seeking behaviour and hostilities decrease. Distance to health facility was another confounder as it affects health seeking behaviour from the one hand, and remote villages far away from any vital infrastructure are less likely to witness war incidents from the other hand.

## Results

### Descriptive analysis

During the period under study, between 1 October 2014 and 30 Jun 2017, 597,675 medical consultations, 68,431 ANC visits, 10,816 deliveries and 4336 C-sections were attended in the studied eight health facilities in northwest Syria. Patients were coming from 11 governorates, 33 districts, 82 sub districts and 882 villages. However, most patients (596,705/597,215: 89·3%) were coming from two governorates, Idleb and Aleppo, where the eight facilities are based. When excluding villages that are more than 15 km away from the closest health facility, the mean distance between villages and closest health facilities was 8·4 km and the median was 8·3 km.

For conflict incidents, our database included information on 11,396 incidents, which took place during the period under study, out of which 10,390 were bombardments, 221 explosions and 785 clashes; and 5849 people were killed in these incidents. These events took place in 515 villages from the same geographical area that is covered by the 8 studied health facilities. 93% of incidents (7674/8272) were bombings which have the highest number of people killed (4184/4545: 92% of total people killed). 60.8% (5027/8272) of the incidents took place in the coverage area of only two health facilities which are the closest ones to front lines. The average number of deaths per incident was 0.55 (ranging between 0 and 104).

**Fig. 1:**
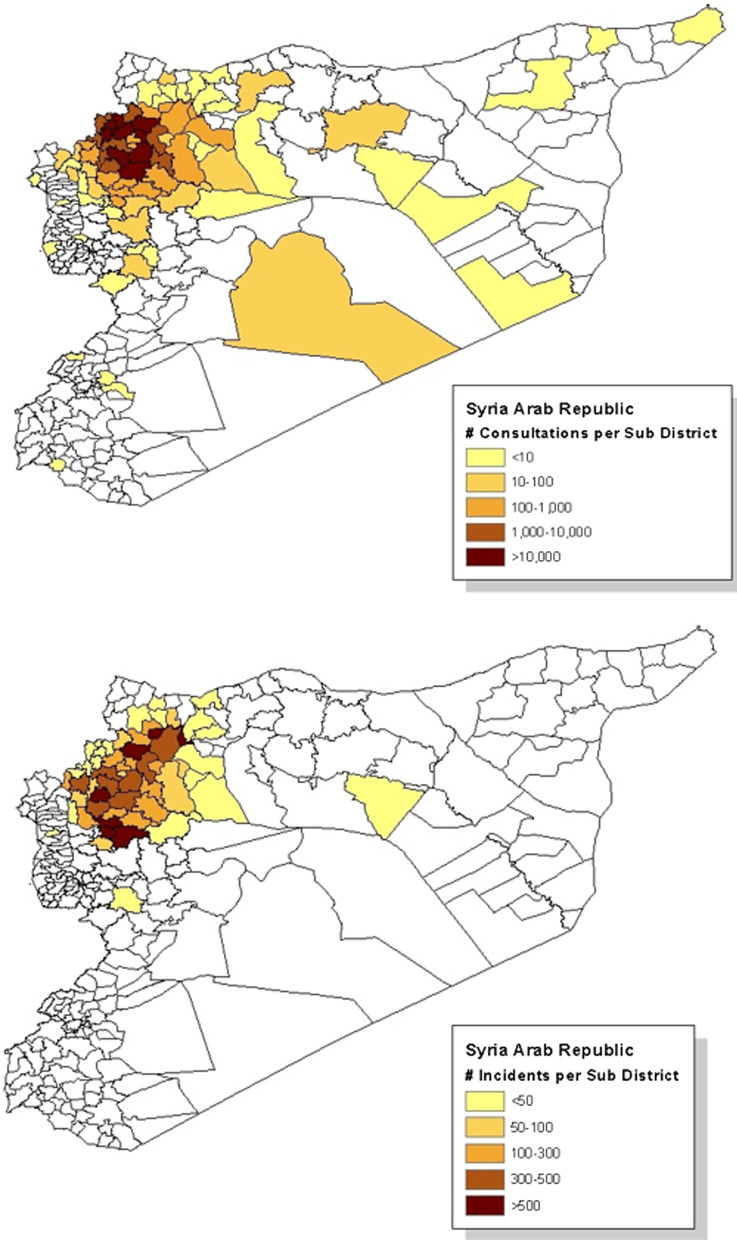
Distribution of the consultations and war incidents across sub districts

**Fig. 2:**
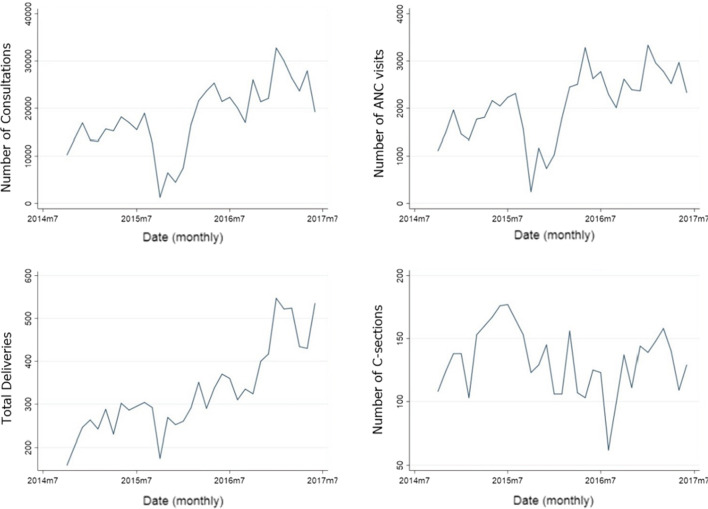
Trends of the outcome variables during the period under study

**Fig. 3:**
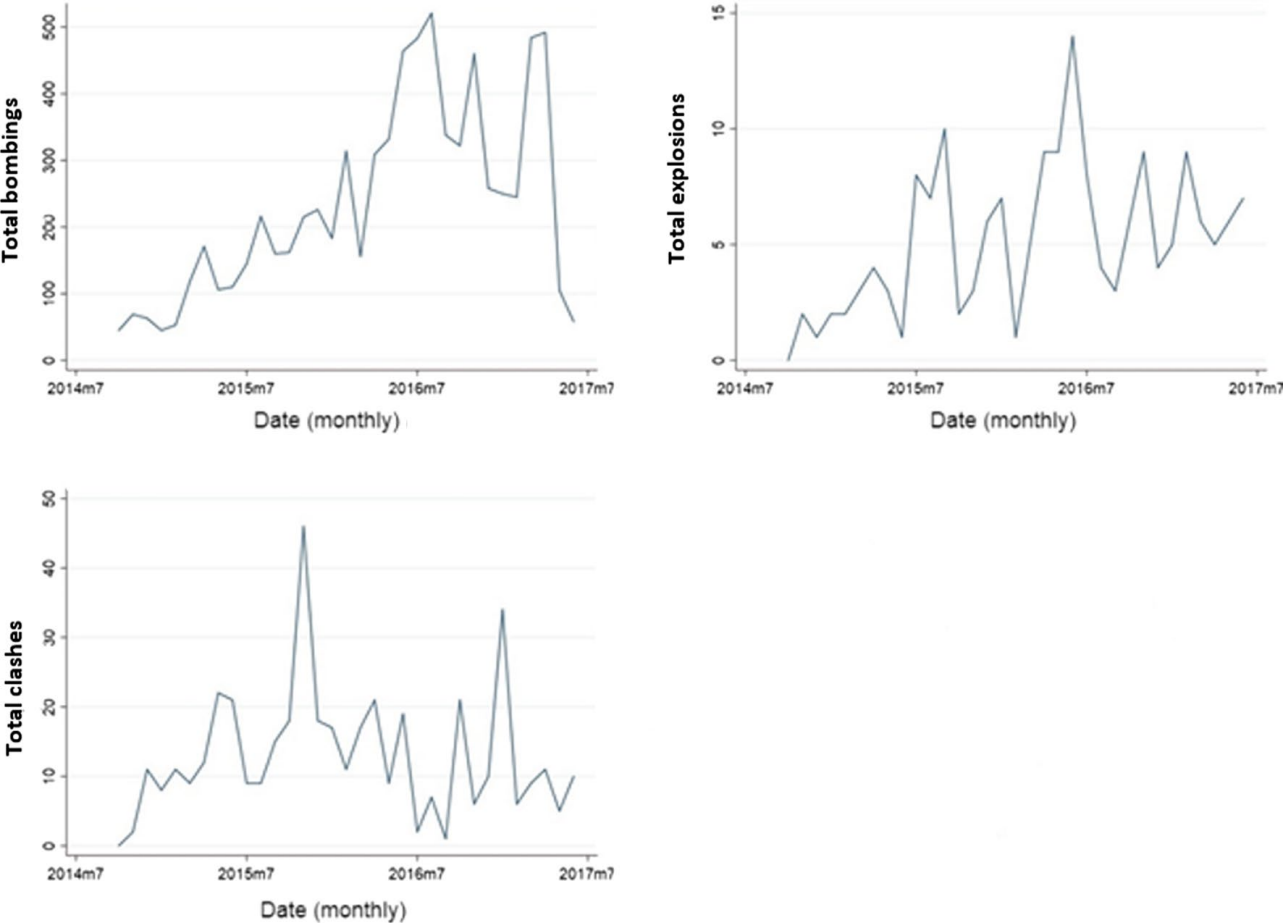
Trends of the exposure incidents during the period under study

When merging the conflict incidents database with the HIS database, only 4323/38% of the incidents took place in villages that were included in the multivariant analysis. The geographical distribution of the consultations and the conflict incidents is shown on the map in Fig. 1. The trends of the studied health indicators and the war incidents over time are shown in Figs. 2 and 3. The drop in all services in October 2015 and the drop in C-sections in August 2016 are explained by the closures of facilities mentioned in the “data inclusion” section.

### Monthly negative binomial model

The study found a strong evidence for a negative association between bombardments and consultations and ANC visits, RR was 0.95 (95% CI 0.94–0.97) and 0.95 (95% CI 0.93–0.98) respectively. These associations were found to be significant across all seasons; however they were found to have more magnitude during winter and fall; RR = 0.85 (95%CI = 0.81–0.90) for consultations during winter, and RR = 0.89 (95%CI = 0.83–0.95) for ANC visits during winter.

The number of explosions was found to be positively associated with deliveries and C-sections. The data provides evidence that each one incident increase in explosions in a given month in a given village was associated with about 20% increase in deliveries and C-sections; RR was 1.22 (95% CI 1.05–1.42) and 1.96 (95% CI 1.03–3.74) respectively. However, these associations were found to be only significant during spring season (Table 1).

### Daily distributed lag model

**Fig. 4:**
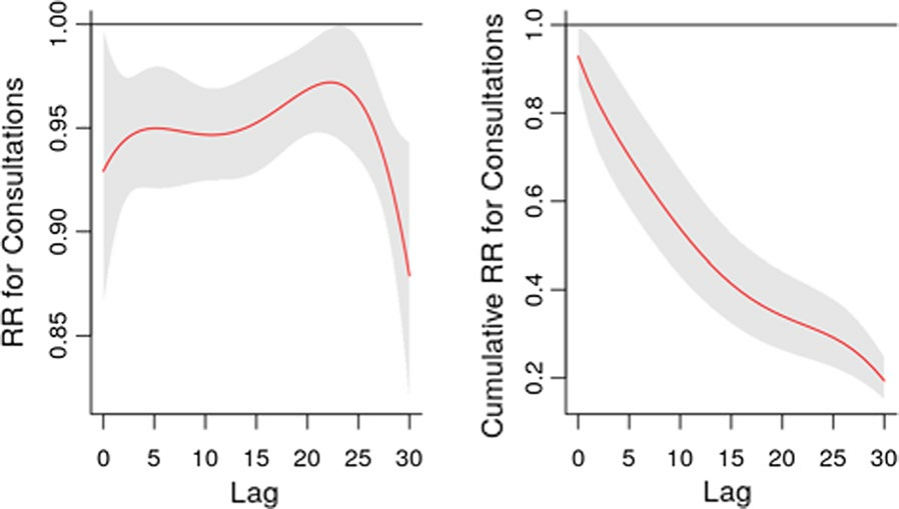
Association of 1 Bombardment on consultations over 30 days

The daily analysis found strong evidence for an association between bombardment incidents and a reduction in consultations over the following 30 days. This association was found to be significant for both the daily RR and the cumulative one (Fig. 4). Whereas both explosions and clashes were associated with an increase in the RR of consultations over the 30 days following incidents. The daily analysis found no other significant associations between any of the outcomes from the one hand and clashes and explosions from the other hand (Table 2).

## Discussion

Our study provides original evidence on the impact of some armed conflict exposures on utilisation of healthcare. This could be caused by either decreased availability of health services, blocked access to health facilities, or changes in the attitude of seeking healthcare by affected population.

Our findings that are related to a negative association between conflict incidents and utilisation of routine health services are in line with various qualitative studies that note a negative link between conflict and utilisation of health services [20–23]. Although, the number of quantitative studies on this topic is limited, we found few studies that reached to similar conclusions to our study. A study in Nepal found a negative correlation between the number of antenatal care check-ups and incidents of conflict-related violence. They found that women are less likely to access ANC during high intensity conflict [24]. Similarly, three other studies in northeast Nigeria, northern Uganda and Liberia found a reduced utilisation of maternal health care services in the areas with high intensity of conflict incidents [25–27]. Another study in Mexico found a decrease in accessing emergency obstetric care while most births were occurring at home during the Zapatista armed conflict [28]. Similar negative correlation was found in a nested case–control study that investigated the utilisation of mental health services before, during and after the Israeli-Lebanese war in 2006. The study found a decline in primary health consultations as well as the utilisation of mental health services during the war [29]. However, most these studies are context-specific and rely either on relatively small sample sizes or on a national level health data such as Demographic and Health Surveys (DHS) with no unique identifier for each patient/consultation.

Our estimated the coverage of eight health facilities in north west Syria in the period between 1 October 2014 and 31 Jun 2017. The catchment area was found to be overstretched and extended to remote communities which, in stable conditions, might not seek healthcare in these facilities. This factor could be a leading factor behinds health seeking behaviour and limited utilisation of health services. Because of the ongoing conflict in Syria, access for healthcare has been limited. People in affected areas, had to travel long distances to access healthcare. These findings are consistent with several reports from medical organisations and personnel in Syria stating lack of access to healthcare as a key need in the Syrian crisis [30, 31]. Reasons behind this include the collapse of the health system in the opposition-controlled areas, poorly resourced humanitarian response and ongoing targeting of health infrastructure in Syria [13, 32–35]. Our findings suggest that the health humanitarian response in Syria do not meet the minimum standards required for health service delivery as set out by relevant guidance such as the Sphere Guidelines on the “Humanitarian Charter and Minimum Standards in Disaster Response” and the UNHCR emergency handbook. For example the Sphere guidelines states that 80% of population should be able to access healthcare within one hour walk, and that there should be at least one primary health care facility per 10,000 people [17, 36].

The study provides evidence for associations between several types of war incidents and the utilisation of key health services. For health seeking behaviour related to routine healthcare, such as out-patient consultations, conflict incidents tend to have a negative impact on the utilisation of these services. Bombardment incidents were found to be associated with decreased consultations and ANC visits. This could be due to a decreased access to healthcare during and following bombing incidents. Some of the SCI-supported health facilities were closed during intensified bombardments. Two of these facilities were hit by airstrikes in the period under study and were closed in the subsequent days.

For health seeking behaviour related to emergency care and labour care, our study suggest a positive association between conflict incidents and the utilisation of these services. Explosions were found to be associated with increased deliveries and C-sections. One explanation might be related to the psychological trauma associated with explosions which could induce labour [37]. Moreover, explosions, and other types of war events, might make women worried about the timings of their deliveries. In such cases, women might prefer to have a planned C-section to avoid unpredictable war conditions during their labour. The same argument can be applied to other types of conflict incidents; however, our studies was not able to find other relevant significant associations.

Accordingly, the study findings can inform related policies that concern design of health interventions and allocation of health resources in conflict settings as well as advocacy and accountability measures.

## Limitations

First, like most natural experiments, the study is prone to lack of ability to control various elements related to the settings and the data quality, such as data collection, the design of the health services in question, and the role of other healthcare providers. The SCI-HMIS has passed through several phases of upgrades, sometimes with different tracked morbidities or different case definitions. This has limited our ability to explore the utilisation of other health services. Furthermore, this HMIS data is limited to only eight health facilities which does not represent all healthcare provided in opposition-controlled areas of north west Syria. Populations living in in any village in the study might have had access to other health services during the period under study.

Secondly, for the war incidents data, we relied on only two local news agencies, and due to time and resources limitations, we could not strictly verify all incidents. We confined the verification to only incidents with casualties. And as clashes and explosion incidents were less frequent in the study population, the study might have lacked the power to detect associations with clashes and explosions in the final models.

Thirdly, the study might be prone to selection bias as the study population covers the communities that are in the coverage area of the SCI-supported health facilities. Those communities might have better access to healthcare than other villages which did not appear in the HMIS data.

Another source of selection bias is that those communities were being served by other health providers in addition to the Save the Children’s project. We tried to mitigate the impact of this bias through including data only from villages with high utilisation of health care services in the studied facilities. These villages tend to be close to the studied health facilities and have limited access to other major health providers. Additionally, the study period, which is about three years, allowed to mitigate this bias considering that Save the Children’s project was one of very few other projects that were sustained in these areas throughout this period as was reported by Save the Children and their implementing partners.

## Conclusion and recommendations

The study has found the coverage of the eight studied facilities to be overstretched to cover wide geographical areas. Some patients were coming from remote places located hundreds of kilometres away from the health facilities. This raises the importance of meeting the minimum standards of humanitarian interventions in the Syrian response.

The study provides evidence for the detrimental impact of armed conflict on the utilisation of primary healthcare services. This detrimental impact might lead to interruption of treatments and accordingly to an increased burden of diseases. For example, women who are less likely to visit health facilities to monitor their pregnancy during active exposure to conflict incidents, are at risk of developing pregnancy-related complications. Also, these associations might reflect a reduced access to healthcare due to either lack of availability of health services or changes in the healthcare seeking behaviour among the conflict affected population.

To tackle some of these consequences, the study suggests few considerations for healthcare providers in conflict settings to ensure availability and accessibility of health services in conflict settings. Allocation of health services and mapping of health facilities should be decentralised with a field central coordination. This can be done through identifying roles and responsibilities between funders and implementers of health programming from the one hand, and local health bodies and authorities, such as the Health Directorates in the case of northwest Syria, from the other hand. For example, each of the studied health facilities used to have its own management structures and practices linked to the central coordination of the project network. In addition, the whole network was linked to the central health body in Idlib governorate, which is Idlib Health Directorate.

Additionally, health actors should ensure the availability of essential health services at the appropriate geographical levels. For example, based on the study findings, BEmOC services should be available in each sub district, while CeMOC services can be available in each district with active referral function between these two levels of services. Also, an appropriate referral system should be put in place between the several levels of healthcare. Another consideration is outreach programming, such as community health workers and mobile clinics, which could be used to address behavioural changes in healthcare seeking attitudes among conflict affected populations as well as addressing key barriers to accessing healthcare.

During the period under study, two of the health facilities were hit by airstrikes and shelling. There is a pressing need to provide more protection for healthcare in conflict settings and to set up additional measures to monitor the adherence and compliance to the relevant legal frameworks such as the Geneva Conventions and United Nations Security Council Resolution 2286 which condemns attacks on healthcare in conflict [38–40].

Finally, there is a large space for further epidemiological research to study conflict impact on health. Such studies can guide the design of health intervention in emergencies, and help to influence the advocacy for human rights, international humanitarian law and protection of civilians during conflict.

**Table 1:** The adjusted RR of 1 unit of each exposure

Exposure	# Consultations		# ANC visits		#Deliveries		# C-Sections	
	Adjusted* RR (95%CI)	P	Adjusted* RR (95%CI)	P	Adjusted* RR (95%CI)	P	Adjusted* RR (95%CI)	P
Bombing	**0.95 (0.94–0.97)**	**< 0.001**	**0.95 (0.93–0.98)**	**< 0.001**	0.99(0.96–1.02)	0.644	1.02 (0.97–1.08)	0.376
Explosion	0.92 (0.82–1.03)	0.152	0.89 (0.78–1.03)	0.218	**1.22 (1.05–1.42)**	**0.008**	**1.19 (1.02–1.40)**	**0.026**
Clashes	1.07 (0.98–1.17)	0.118	1.06 (0.95–1.19)	0.273	1.15 (0.99–1.33)	0.075	0.96 (0.78–1.18)	0.697

The estimates in bold are the significant ones that we are 95% sure they reflect the real impact and are not due to random chance (*P* < 0.05)

*RR is adjusted for all other variables in the table, Ramadan, season and distance to closest health facility

**Table 2:** The cumulative RR of 1 unit of each exposure at 30 days

Exposure	# Consultations	# ANC visits	#Deliveries	# C-Sections
	Cum. RR (95% CI)	Cum. RR (95% CI)	Cum. RR (95% CI)	Cum. RR (95% CI)
Bombing	0.19 (0.15–0.25)	0.42 (0.25–0.69)	0.12 (0.04–0.41)	0.00 (0.00–0.09)
Explosion	106.75 (26.66–427.48)	464.58 (26.34–8195.32)	109.48 (0.18–65,414.85)	0.49 (0.00–11,326,499.73)
Clashes	28.25 (4.45–179.23)	1.45 (0.03–66.77)	3.58 (0.00–3981.40)	1367.95 (0.01–213,578,839.48)

## Data Availability

The authors had written consents to use the Health Information Management System (HMIS) from both Save the Children International and from their implementing partner (Syria Relief).
